# Palmitoylation Depth Determines Lipid-Phase Preference in Claudins

**DOI:** 10.1021/acs.langmuir.6c03476

**Published:** 2026-10-29

**Authors:** Xuyang Qin, Shikha Nangia

**Affiliations:** Department of Biomedical and Chemical Engineering, 2029 Syracuse University, Syracuse, New York 13244, United States

## Abstract

S-palmitoylation is a reversible lipid modification that regulates membrane protein organization and function, but its molecular consequences remain incompletely understood. Claudins, the primary functional proteins of tight junctions, contain conserved palmitoylation sites on the cytosolic leaflet that influence tight junction assembly and barrier function. Using MARTINI coarse-grained molecular dynamics simulations, we investigated how palmitoylation affects claudin-lipid interactions and membrane phase partitioning in four claudins (CLD3, CLD4, CLD9, and CLD14) in phase-separated bilayers. Palmitoylation enhances claudins’ affinity for ordered phases, promotes local cholesterol enrichment, and reduces disordered-lipid contacts. We find that the membrane-embedded depth of the palmitoyl chain is a key determinant of this preference: sites closer to the membrane interface stabilize claudins within ordered domains, while deeper sites favor disordered environments. This depth dependence arises from differential palmitoyl chain tilting, with upright packing at shallow sites and forced lateral tilting at deeper sites. These results identify palmitoylation depth as a structural determinant of claudin-lipid interactions and provide insight into how this reversible modification governs tight junction protein localization within heterogeneous membranes.

## Graphical abstract

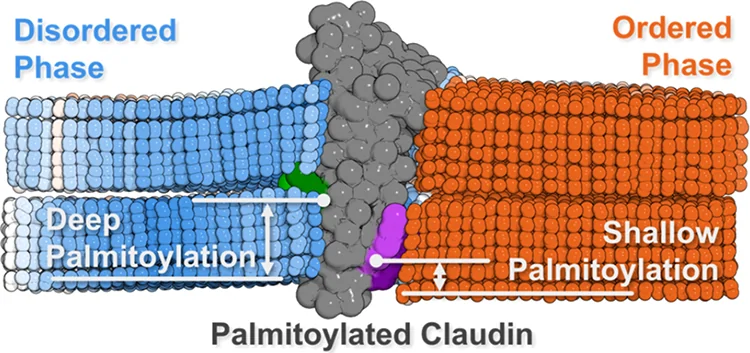

## Introduction

Modification of proteins with fatty acids as in S-palmitoylation is ubiquitous in eukaryotic cells.^1–5^ S-palmitoylation, the addition of palmitic acid (16:0) to a cysteine residue via a thioester bond, is reversible, unlike other lipid modifications. This feature gives palmitoylation a unique ability to regulate protein dynamics and biological function.^6^ In peripheral proteins, the hydrophobic palmitoyl chain temporarily anchors the lipoprotein to the membrane. In contrast, palmitoylation of membrane proteins occurs only at cysteine residues in cytosolic loops or transmembrane domains juxtaposed to the cytosolic membrane interface (Figure 1). While palmitoylation alters protein–protein and protein–lipid interactions, leading to changes in protein function—including receptor signaling,^7–9^ modified trafficking,^10–14^ reduced transporter activity,^15–18^ and ion-channel regulation^19–21^ —how specific palmitoylated sites impact a protein’s function requires further investigation.

**Figure 1:**
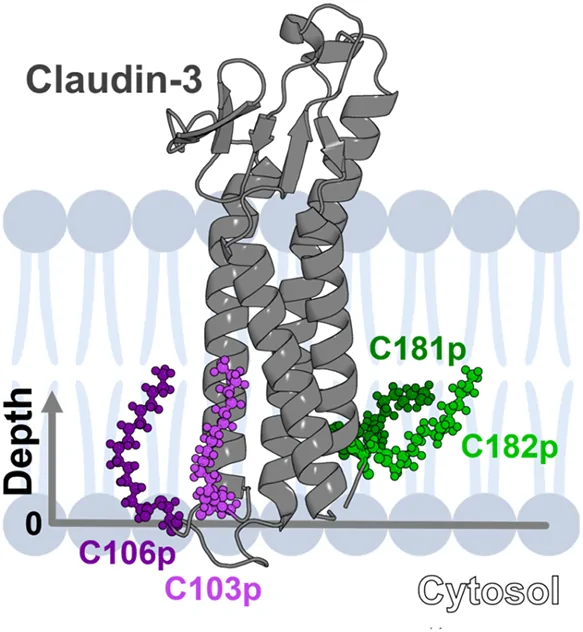
Schematic of palmitoylated claudin-3 embedded in lipid bilayer. The four palmitoylated cysteine residues (C103p, C106p, C181p, and C182p) are organized into two nearby pairs: C103p and C106p are on the intracellular loop, the C181p and C182p are on the fourth helix near C-ter. They exhibit different membrane embedded depths within the cytosolic leaflet.

Advances in our understanding of the structural and biophysical consequences of palmitoylation on membrane protein dynamics and function have been constrained by multiple biochemical and technical factors. These include (a) reversibility of the thioester bond: the rapid attachment and detachment of palmitoyl chains make detection of palmitoylated proteins arduous;^5,22,23^ (b) lack of consensus sequence: a conserved amino acid sequence motif for palmitoylation lacks confirmation;^3,5,24^ (c) degree of palmitoylation: membrane proteins often contain multiple cysteines that can be palmitoylated concurrently, and some studies suggest that a protein can acquire up to four palmitoyl chains;^25^ (d) hydrophobicity of membrane proteins: the relatively high hydrophobic character of membrane proteins makes them difficult to isolate without detergents;^26^ and (e) complex membrane environment: membrane proteins are embedded in lipid environments that exhibit wide chemical and compositional diversity, making protein–lipid interactions harder to decipher.^27–29^ Thus, while current experimental approaches can detect the presence of palmitoyl chains, they cannot characterize at a molecular level the biophysics of a palmitoylated protein—information that is critical to determining its dynamics and function.

A key consequence of palmitoylation in membrane proteins is its ability to direct protein partitioning into distinct lipid microdomains.^11,13,14^ Cellular membranes are laterally heterogeneous, exhibiting coexisting ordered and disordered lipid phases. Ordered domains, enriched in saturated phospholipids and cholesterol, are commonly referred to as lipid rafts, while disordered regions are enriched in unsaturated lipids. Palmitoylation has been proposed to promote protein association with ordered, raft-like domains by increasing the hydrophobic compatibility between the palmitoyl chain and the tightly packed saturated lipid environment.^11,13,14,30–32^ However, membrane proteins often carry multiple palmitoylation sites at different positions along their cytosolic surface, and whether each site exerts an equivalent or site-specific effect on lipid-phase preference remains poorly understood. A central but unresolved question is whether the membrane-embedded depth of a palmitoylation site—its position within the cytosolic leaflet—governs the nature of its lipid interactions and, by extension, the protein’s spatial organization within a laterally heterogeneous membrane.

Claudins are a large family of transmembrane proteins serving as core functional components of tight junctions across diverse tissues, where they regulate paracellular permeability and maintain epithelial and endothelial barrier integrity.^33,34^ Claudin proteins self-assemble into strands on the membrane and engage in head-on interactions with claudins from adjacent cells to form tight junctions, which act as size- and charge-selective barriers between neighboring epithelial cells. Palmitoylation of claudins typically occurs on cysteine residues near the cytosolic leaflet of the membrane^25,31,32,35^ and is increasingly recognized as an important regulatory mechanism that fine-tunes claudin trafficking, assembly, and tight-junction function by promoting partitioning into specific lipid microdomains.^25,31,32,35^ Crucially, claudin palmitoylation sites are organized into two conserved cysteine pairs that reside at distinct membrane-embedded depths (Figure 1): one pair located on the intracellular loop, positioned closer to the membrane interface, and a second pair near the C-terminus of the fourth transmembrane helix, which can be inserted more deeply into the hydrophobic core of the cytosolic leaflet. This structural arrangement raises the possibility that the two site pairs impose different lipid-sorting behaviors and orient the protein distinctly with respect to ordered and disordered membrane phases—a question with direct implications for how claudins are organized at tight junction strands.^25,30–32,36^ Testing this possibility directly would require simulating multiple claudin copies undergoing cis-assembly, which is beyond the scope of the present single-protein study; we return to this connection once our depth-dependent findings are established.

In our previous work, we investigated the effect of palmitoylation on claudin-5,^30^ a critical component of the tight junctions in vascular endothelial cells that are essential for maintaining the blood-brain barrier. At both atomistic and coarse-grained scales, we showed that palmitoylation impacts claudin-lipid and claudin–claudin pair interactions across different lipid compositions. Notably, palmitoylated claudin-5 proteins were found to localize at the boundary between ordered and disordered lipid domains in a phase-separated membrane model, indicating that palmitoylation promotes positioning at membrane phase boundaries rather than exclusive association with a single lipid phase. The presence of two cysteine pairs at distinct membrane depths motivated us to ask whether depth itself is a structural determinant of lipid-phase regulation. Because a single-protein study cannot establish whether such a relationship reflects a general structural principle or a feature specific to claudin-5, we extended this question to four claudin family members spanning a range of sequence identities and depth separations, allowing us to test whether palmitoylation depth is a conserved, generalizable determinant of claudin-lipid sorting rather than a protein-specific effect.

Here we investigate four claudin family members—claudin-3 (CLD3),^37–39^ claudin-4 (CLD4),^31,40–42^ claudin-9 (CLD9),^43–45^ and claudin-14 (CLD14)^25,46,47^ —which share varying degrees of sequence similarity while retaining the conserved two-pair palmitoylation site architecture (Table 1).^25,35^

**Table 1:** List of Human Claudin Proteins Investigated in this Work

Claudin	UniProt	Structure Source	Palmitoylation Sites
CLD3	O15551	AlphaFold2(v2.3.2)	C103, C106, C181, C182
CLD4	O14493	PDB: 7KP4 ^42^	C104, C107, C182, C183
CLD9	O95484	PDB: 6OV2 ^44^	C104, C107, C182, C183
CLD14	O95500	Homology Modeled	C104, C107, C182, C185

Using 10-microsecond coarse-grained molecular dynamics simulations in ternary phase-separated lipid bilayers composed of DPPC (ordered), DIPC (disordered), and cholesterol, we demonstrate that palmitoylation depth is a key structural determinant of lipid-phase preference. Shallow palmitoylation sites favor ordered lipid contacts through upright palmitoyl chain packing, while deeper sites adopt tilted chain geometries that favor the disordered phase. This depth-dependent lipid sorting positions palmitoylated claudins at membrane phase boundaries, with implications for tight junction strand assembly and the general organization of palmitoylated proteins in laterally heterogeneous membranes.

## Methods

### Protein Structures

Atomistic structures of the human claudin proteins were obtained from different sources depending on their availability. The CLD4 and CLD9 structures of human claudins were obtained from the RCSB Protein Data Bank (Table 1). Due to the lack of experimental crystal structures for human CLD3 and CLD14, the CLD14 structure was generated by homology modeling of the human sequence in YASARA,^48^ while the human CLD3 structure was predicted using AlphaFold2 ^49^ (v2.3.2). The residues belonging to the unstructured C-terminal tail were not included in any of the systems.

### Atomistic Equilibrations

All atomistic simulations were performed in GROMACS 2023.2.^50^ The CHARMM-GUI Web server^51–55^ was used to palmitoylate the cysteine sites in the proteins and build the solvated protein–membrane systems for atomistic simulation. Each of the nonpalmitoylated and palmitoylated claudins was individually embedded in a 10 × 10 nm^2^ membrane of 1-palmitoyl-2-oleoylphosphatidylcholine (POPC) and solvated in TIP 3p^56,57^ water with 0.15 M NaCl using CHARMM36m^58^ force field parameters. Each system was energy minimized using the steepest descent algorithm with a maximum force tolerance parameter of 10^3^ kJ mol^–1^ nm^–1^, followed by a 2 ns isothermal-isochoric (NVT) and a 2 ns isothermal–isobaric (NPT) equilibration, respectively. Position restraints were applied to the heavy atoms in proteins and lipids during the NVT using a force constant of 400 kJ mol^–1^ nm^–2^, which was reduced to 40 kJ mol^–1^ nm^–2^ during NPT equilibration. Lipids were restrained only on phosphorus atoms for *z* direction. The time step of the simulations was set to 2 fs. The electrostatic and van der Waals interactions cutoff was set as 1.2 nm. Force-switch was applied to smoothly switch the Lennard–Jones interactions starting at 1.0 nm. The Particle-Mesh Ewald (PME)^59^ method was applied for long-range electrostatic interactions. The temperature of the simulations was maintained at 303 K using a v-rescale^60^ with a temperature coupling constant of 1.0 ps. The hydrogen bonds were constrained using the linear constraint solver (LINCS) algorithm. In the NPT equilibration simulations, the semi-isotropic pressure coupling was maintained at 1 bar pressure using the Berendsen^61^ with a coupling constant of 5.0 ps and compressibility constant of 4.5 × 10^–5^ bar^–1^. The production runs were performed under NPT settings for 100 ns, while all position restraints were removed and Parrinello–Rahman^62^ barostat was used. Other simulation parameters were same as the prior NPT equilibration step. These atomistic production runs served as the source of equilibrated protein structures for subsequent coarse graining; no atomistic trajectory analysis was performed.

### Coarse-Grained Simulations

The protein structures from the atomistic equilibrations were coarse-grained under MARTINI 2.2 force field in combination with the elastic network (ElNeDyn22) using the *martinize.py* script.^63^ For palmitoylated cysteine residues, we followed the parameters constructed in previous work.^30^ The claudins were individually placed in the lipid bilayers with three components: 1,2-Dipalmitoyl-*sn*-glycero-3-phosphocholine (DPPC, an ordered lipid), 1,2-dilinoleoyl-*sn*-glycero-3-phosphocholine (DIPC, a disordered lipid), and cholesterol (CHOL). Using the *INSANE* script,^64^ each protein was inserted into a 15 × 15 nm^2^ membrane patch initially containing a homogeneous mixture of DPPC, DIPC, and CHOL at a molar ratio of 5:3:2.^14^ The lipid composition was selected based on established ternary phase diagrams that exhibit stable liquid-ordered and liquid-disordered phase coexistence at 303 K, providing a physiologically relevant model of membrane lateral heterogeneity. During equilibration, the lipids reorganized and underwent phase separation into coexisting liquid-ordered and liquid-disordered domains, progressing from the initial homogeneous mixture to the final phase-separated state (Figure S7). The systems were solvated using MARTINI 2 waters (W:WF = 9:1) and 0.15 M NaCl as a 15 × 15 × 15 nm^3^. The steepest descent algorithm was used for energy minimization with a maximum force tolerance parameter of 100 kJ mol^–1^ nm^–1^. NVT and NPT equilibrations were performed for 10 and 40 ns, respectively. During NPT and NVT steps, position restraints were applied to the protein backbone beads. Following the standard MARTINI 2.2 simulation settings, the 20 fs time step was used. The cutoff for the electrostatic and van der Waals interactions was set as 1.1 nm, and the reaction-field method was used for the electrostatic interactions with epsilon-r = 15 and epsilon-rf = 0. The temperature of 303 K was maintained using the v-rescale thermostat with a temperature coupling constant of 1.0 ps. The lipid membrane, protein, and solvent were set as different groups for temperature coupling. In the NPT simulations, semiisotropic pressure coupling was maintained at 1 bar using the Berendsen barostat with the coupling constant of 5.0 ps and compressibility constant of 3 × 10^–4^ bar^–1^. The production runs were performed for 10 μs under NPT conditions using the same settings as the prior NPT equilibration step, except all position restraints were removed and Parrinello–Rahman barostat was used with 12 ps coupling constant.

### Analysis

Trajectories from 4–10 μs were extracted for analysis. All trajectories were least-squares fitted to the protein centered in the simulation box. Lipid enrichment along the trajectory in the cytosolic leaflet, as well as trajectory-averaged structures of the membranes and proteins, were analyzed and generated using the MOSAICS^65^ suite. In-house scripts based on the MDAnalysis^66^ package were used to calculate the normalized lipid contact probability and the embedded depth for the palmitoylation sites along the trajectories; the underlying principles and mathematical details are described in the Supporting Information (S1 and S2). The trajectory-based data were block-averaged using the GROMACS suite with five or six blocks to obtain the total mean value and block-averaged standard error. The *z*-axis order parameters (*S*_*zz*_) of the palmitoyl chains were computed for the side chains of palmitoylated cysteines using GROMACS, and subsequently averaged and converted to palmitoyl chain tilt angles as illustrated in Supporting Information S3.

## Results and Discussion

### Palmitoylation Sites Exhibit Distinct Membrane Insertion Depths across Claudin Family Members

The sequence alignment and membrane-embedded depth analysis presented in Figure 2 establish the structural architecture of claudin palmitoylation sites. We show that the palmitoylation site architecture is conserved at the sequence level, yet the precise membrane-embedded depths vary in a consistent shallow-deep pattern that differs quantitatively across claudin family members.

**Figure 2:**
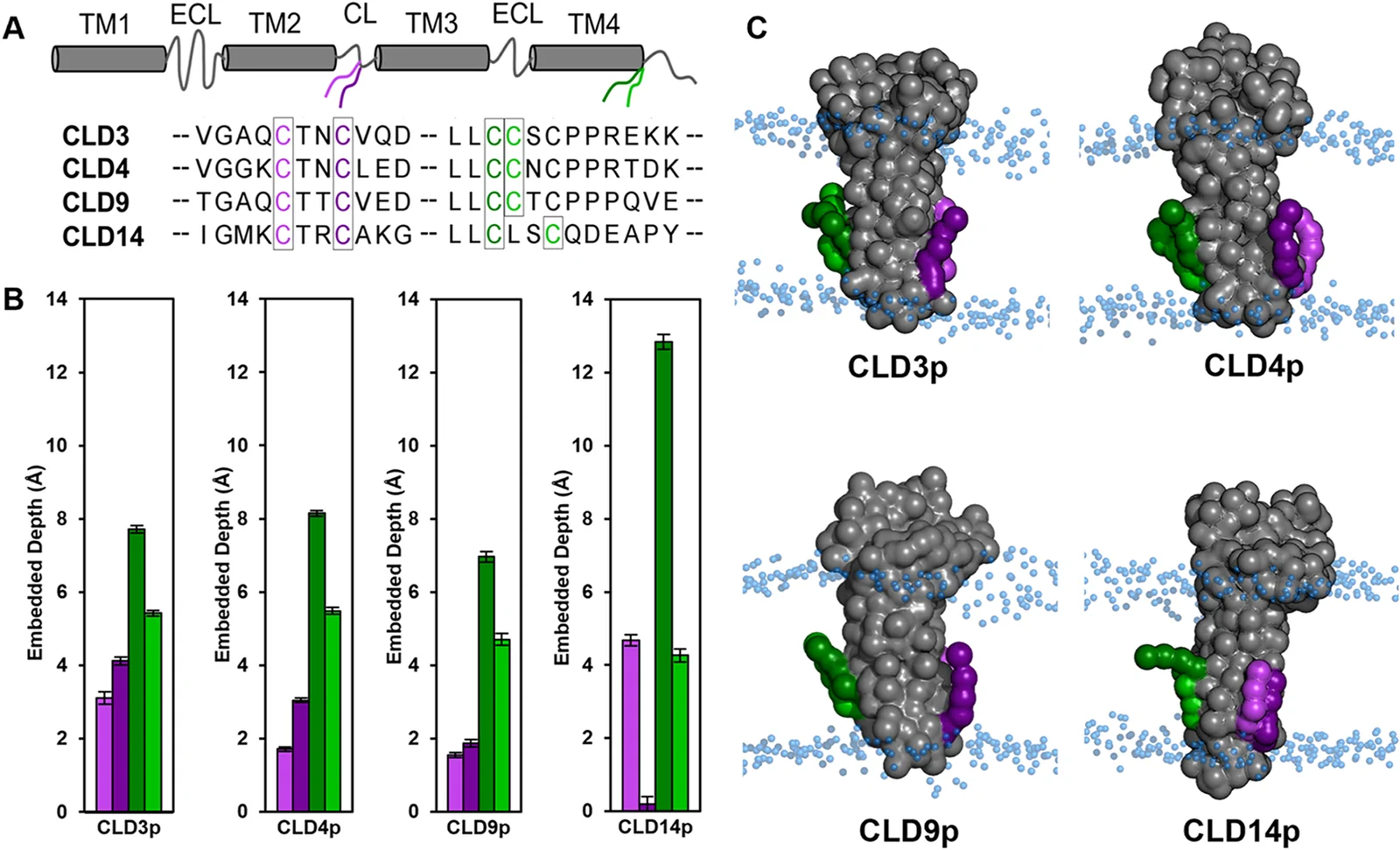
(A) Sequence alignment near the two pairs of palmitoylation sites. TM1–TM4, transmembrane helices; ECL, extracellular loop; CL, cytosolic (intracellular) loop. (B) Membrane-embedded depths of palmitoylated cysteine residues in CLD3, CLD4, CLD9, and CLD14. Colors correspond to the positions shown in A, with lighter and darker shades distinguishing residues within each pair. Embedded depths are referenced to the cytosolic membrane surface. Error bars represent block-averaged standard errors. (C) Representative snapshots of palmitoylated claudins in the phase-separated membrane. The transparent blue beads represent the membrane surface defined by the PO4 beads.

The sequence alignment (Figure 2A) reveals that all four claudins possess two pairs of palmitoylatable cysteines at topologically equivalent positions: one pair on the intracellular loop between TM2 and TM3, and a second pair at the junction of TM4 and the C-terminal tail. Within each pair, the two cysteines are separated by zero to two residues, and the flanking sequence context is broadly conserved, particularly around the CC or CxxC motif. This conservation suggests that the spatial geometry of palmitoylation, two closely spaced chains anchored at distinct membrane depths, is a shared architectural feature of the claudin family. The conservation also implies that the depth asymmetry observed in simulations is encoded in the primary sequence and transmembrane topology, not an artifact of any particular structural model.

The depth measurements in Figure 2B quantify this asymmetry across all four claudins. In every case, the intracellular loop cysteine pair (shown in purple) resides at a shallower membrane-embedded depth of approximately 0–5 Å from the cytosolic leaflet interface, while the TM4 cysteine pair (shown in green) penetrates substantially deeper into the hydrophobic core of the cytosolic leaflet, reaching depths of 4–13 Å depending on the claudin. Detailed depth values for all sites are provided in Supporting Information S5.

The magnitude of depth separation between each claudin’s two cysteine pairs, calculated as the difference between the mean depth of the shallow (intracellular loop) pair and the mean depth of the deep (TM4) pair, also differs across claudins. CLD14 exhibits the greatest depth separation between its two pairs, with the deep pair reaching approximately 12–13 Å, the largest membrane penetration observed among all residues examined. CLD4 and CLD9 show intermediate depth separations, and CLD3 exhibits the smallest depth separation between its two pairs among the four claudins examined.

Native mass spectrometry studies have found that cysteine palmitoylation *in vivo* is generally restricted to residues within about 8 Å of the membrane interface, reflecting the limited access of the palmitoyl-acyltransferase enzyme to more deeply buried cysteines.^35^ The shallow, intracellular-loop cysteine pair falls within this range for all four claudins. The deeper TM4 C-terminal pair also falls within this range for CLD3, CLD4, and CLD9, but exceeds it for CLD14, whose deep pair reaches approximately 12–13 Å in our simulations. This discrepancy may reflect a difference in true palmitoylation accessibility for CLD14 not captured by our model, and we note it as a limitation of the CLD14 system specifically.

The relationship between sequence conservation and depth variability also raises an important structural point. While the CC or CxxC palmitoylation motif is conserved, the surrounding residue context, particularly the identity of residues between the two cysteines in each pair and the local secondary structure at the TM4-C-terminal junction, differs across claudins and likely contributes to the quantitative variation in embedded depth. This suggests that the depth of palmitoylation could be tuned by sequence variation within a conserved topological framework.

Thus, Figure 2 establishes that claudin palmitoylation sites are organized into a conserved two-tier depth architecture, shallow interfacial sites and deep hydrophobic core sites, with insertion depth varying quantitatively across family members. We next examine whether, and how, this depth architecture shapes each claudin’s interactions with the surrounding lipid environment.

### Palmitoylation Increases Claudins’ Affinity for the Ordered Lipid Phase

The lipid-phase behavior discussed below relies on MARTINI’s well-established ability to reproduce liquid-ordered/liquid-disordered coexistence in ternary DPPC/DIPC/cholesterol membranes, a lipid–lipid property distinct from MARTINI 2.2′s known tendency to overestimate protein self-association in systems containing multiple protein copies.^67,68^ Because each system here contains a single claudin monomer, this aggregation artifact does not apply; recent refinements in MARTINI 3 ^69^ improve the quantitative sharpness of lipid gel–fluid transitions but do not indicate a qualitative failure of MARTINI 2.2 to capture the phase separation relied upon here. We also note that this ternary system is a simplified representation of native tight-junction membranes, which are compositionally more complex and asymmetric between leaflets. Cholesterol and long-chain sphingomyelin, both ordered-phase-promoting lipids, are specifically enriched at tight junctions *in vivo* and required for barrier function,^70^ and apical membrane compartments in polarized epithelial cells are independently measured to be cholesterol-enriched and more biophysically ordered than basolateral membrane,^71^ supporting the physiological relevance of an ordered/disordered axis of variation, though the precise lipid composition of the tight-junction membrane itself remains undefined.

The presence of palmitoyl chains on claudins significantly alters the local lipid environment surrounding the protein. In the absence of palmitoylation, all four claudins show a clear tendency to localize within the DIPC-rich disordered membrane region, as shown by the blue-dominated lipid enrichment maps surrounding each protein in Figures 3 and 4. Upon palmitoylation, the lipid enrichment maps reveal a substantial redistribution of each claudin toward the DPPC-enriched ordered domain, with the palmitoylated proteins repositioning at the interface between the ordered and disordered lipid phases. This trend is further supported by the lipid contact probability analysis at each palmitoylated cysteine residue before and after modification, which consistently shows increased interactions with ordered-phase lipids—particularly DPPC and CHOL—and reduced association with the disordered lipid DIPC across all claudins examined. Detailed lipid contact probability values for all residues and conditions are provided in Supporting Information S4.

**Figure 3:**
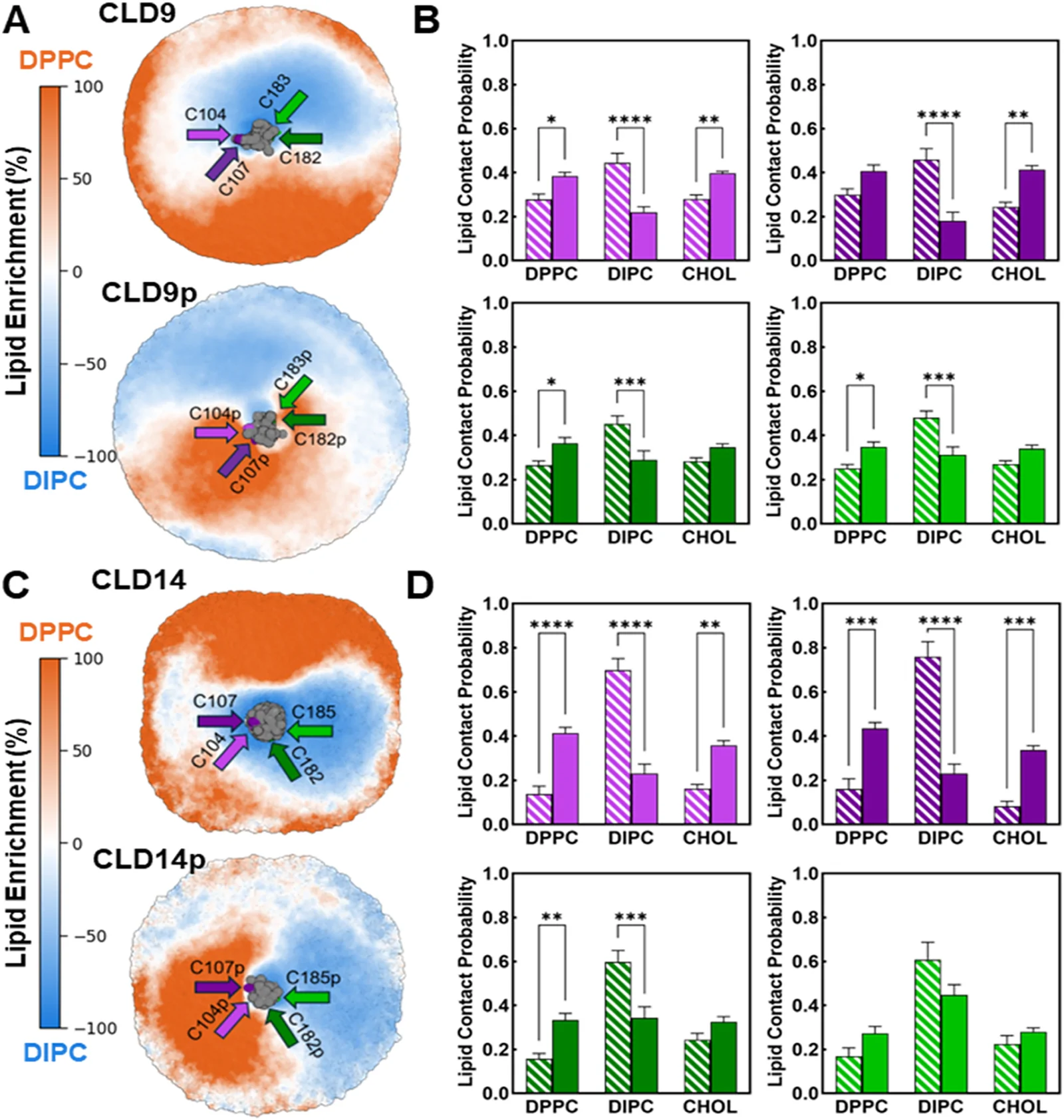
Palmitoylation significantly enhances CLD9 and CLD14 preference for the ordered lipid phase. (A, C) Lipid enrichment maps of the cytosolic leaflet before (top) and after (bottom) palmitoylation. DPPC (orange) and DIPC (blue) distributions are shown around the protein (gray); palmitoylation sites are highlighted as purples and greens. The circular shape of the lipid enrichment maps results from the rotational alignment of trajectory frames during the averaging procedure and does not represent the geometry of the simulated membrane. (B, D) Lipid contact probabilities at each palmitoylated cysteine residue before (hatched) and after (solid) palmitoylation, colored by residue identity as in panels (A, C). Error bars indicate the block-averaged standard errors. Two-way ANOVA was used to assess the statistical significance of change for each lipid type on each cysteine site independently, indicated as follows: no label (not significant, *p* > 0.05), *­(*p* ≤ 0.05), **­(*p* ≤ 0.01), ***­(*p* ≤ 0.001), and ****­(*p* ≤ 0.0001).

**Figure 4:**
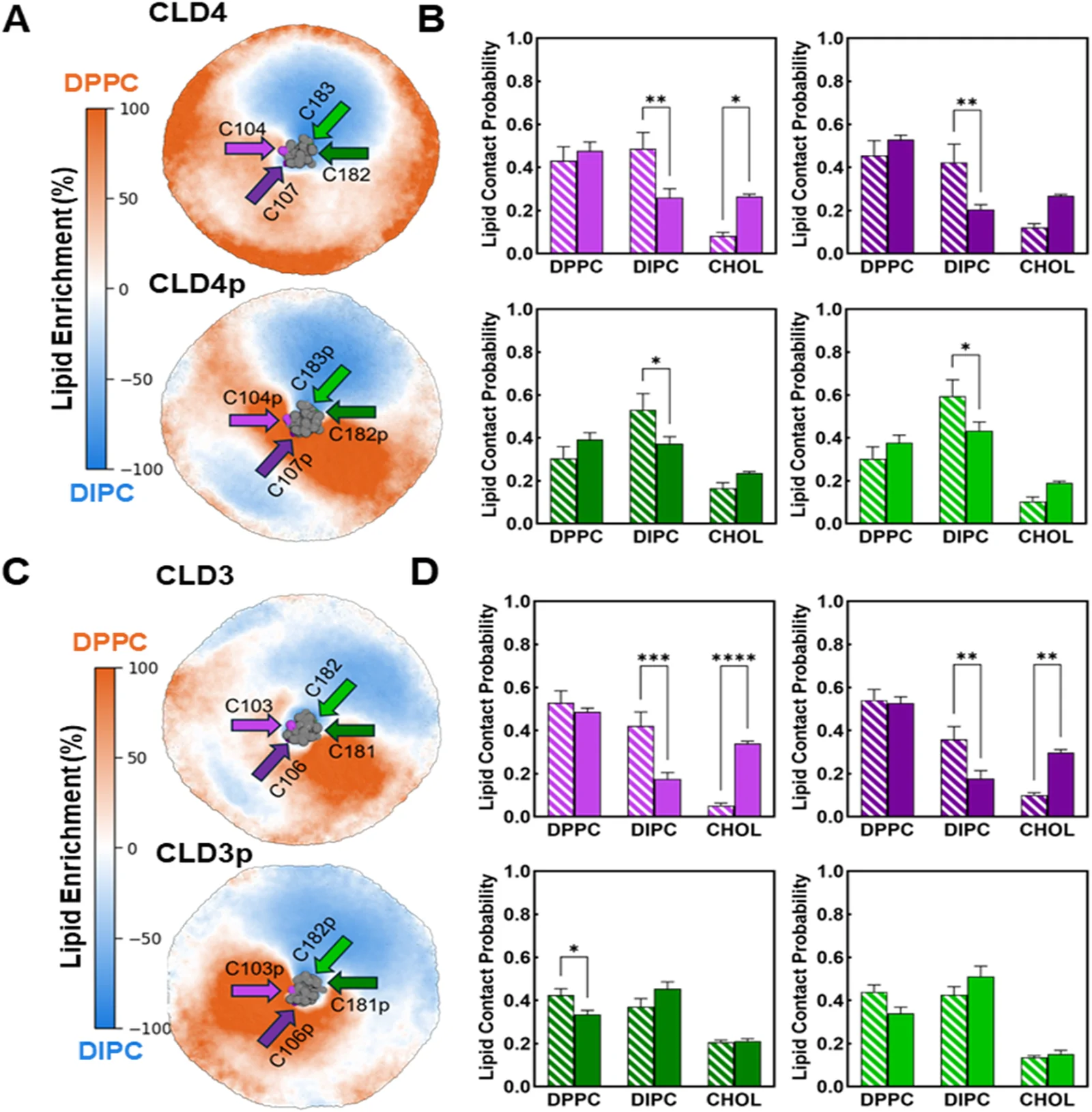
Palmitoylation significantly enhances CLD4 and CLD3 preference for the ordered lipid phase. (A, C) Lipid enrichment maps of the cytosolic leaflet before (top) and after (bottom) palmitoylation. DPPC (orange) and DIPC (blue) distributions are shown around the protein (gray); palmitoylation sites are highlighted as purples and greens. The circular shape of the lipid enrichment maps results from the rotational alignment of trajectory frames during the averaging procedure and does not represent the geometry of the simulated membrane. (B, D) Lipid contact probabilities at each palmitoylated cysteine residue before (hatched) and after (solid) palmitoylation, colored by residue identity as in panels (A, C). Error bars indicate the block-averaged standard errors. Two-way ANOVA was used to assess the statistical significance of change for each lipid type on each cysteine site independently, indicated as follows: no label (not significant, *p* > 0.05), *­(*p* ≤ 0.05), **­(*p* ≤ 0.01), ***­(*p* ≤ 0.001), and ****­(*p* ≤ 0.0001).

CLD9 and CLD14 exhibit the most pronounced effect, with palmitoylation almost completely reversing their initial preference for the disordered phase (Figure 3). The palmitoylation sites in CLD9—C104, C107, C182, and C183—and in CLD14—C104, C107, C182, and C185—are marked by purple and green arrows in the cytosolic leaflet (Figure 3A,C), with the purple intracellular loop pair positioned shallower and the green C-terminal pair inserted more deeply. Upon palmitoylation, the lipid enrichment maps undergo a striking transformation for both claudins. The protein transitions from a DIPC-rich environment to one where DPPC-enriched ordered lipid domains dominate the immediate protein surroundings, with the palmitoylated proteins now situated at the interface between the orange ordered phase and the blue disordered phase. This phase-boundary positioning is particularly pronounced in CLD14p, where the shallow purple palmitoylation sites point toward the ordered phase and the deep green sites orient toward the disordered phase, visually anticipating the depth-dependent mechanism described in the following subsection.

The lipid contact probability data quantify this redistribution (Figure 3B,D). For CLD9p, palmitoylation increases DPPC and CHOL contacts at all four modified cysteines, resulting in marked decreases in DIPC contacts: C104 decreases from 0.44 to 0.22; C107 from 0.46 to 0.18; C182 from 0.45 to 0.29; and C183 from 0.48 to 0.31. The reductions in DIPC contact are statistically significant at all four sites (*p* ≤ 0.001 or better), while increases in DPPC and CHOL contacts reach significance primarily at the shallow C104p and C107p sites, consistent with their greater compatibility with ordered lipid packing. CLD14 shows an even more pronounced reversal of lipid preference. DPPC and CHOL contacts increase substantially at all four sites following palmitoylation, while DIPC contacts decrease markedly: at C104 from 0.70 to 0.23, at C107 from 0.76 to 0.23, at C182 from 0.60 to 0.34, and at C185 from 0.61 to 0.48. The effect is most statistically robust at the shallow purple sites—C104p and C107p—where DPPC and CHOL enrichment reaches *p* ≤ 0.0001, reflecting the tight packing compatibility between upright shallow palmitoyl chains and the ordered phase. At the deeper green sites—C182p and C185p—the increase in ordered-phase contacts is present but more modest, and the statistical significance is correspondingly lower, consistent with the tilted chain geometry that reduces compatibility with tightly packed ordered lipids.

A similar but less complete reversal is observed for CLD4 and CLD3 (Figure 4). For CLD4p, palmitoylation at C104, C107, C182, and C183 increases both DPPC and CHOL contact probabilities while reducing DIPC contacts at all four sites. The reduction in DIPC is greater at the shallow pair—C104 decreasing from 0.49 to 0.26 and C107 from 0.42 to 0.20—than at the deeper pair, where C182 decreases from 0.53 to 0.37 and C183 from 0.59 to 0.43, already foreshadowing the depth-dependent pattern that will be examined in detail in the following subsection. For CLD3, the unmodified protein is surrounded by a mixture of DIPC and DPPC (Figure 4C). Following palmitoylation, there is a clear increase in DPPC enrichment around the protein, particularly near the C103p and C106p regions. Correspondingly, CHOL contacts at C103p and C106p increased significantly from 0.05 and 0.10 to 0.34 and 0.30 respectively, while DIPC contacts decreased from 0.42 and 0.36 to 0.18 for both residues. In contrast, the enhancement of ordered-phase interactions is less evident at the deeper C181p and C182p sites, where changes in DPPC and CHOL contacts are smaller in magnitude and reach lower levels of statistical significance.

Across all four claudins, reductions in DIPC contact probabilities upon palmitoylation were the most statistically robust outcome, reaching *p* ≤ 0.001 at most modified cysteine sites and representing the most consistent signature of palmitoylation across the claudin family. Increases in DPPC contact probabilities were similarly significant at shallow palmitoylation sites—particularly at the intracellular loop cysteine pairs—where ordered-phase enrichment reached *p* ≤ 0.01 or better in CLD3, CLD9, and CLD14. In contrast, changes in DPPC contacts at deeper cysteine sites were more variable, with several comparisons reaching only *p* ≤ 0.05 or falling below the significance threshold, consistent with the weaker ordered-phase affinity observed at these positions. Cholesterol contact changes showed the least uniform significance across claudins. We observe that CHOL enrichment was statistically significant at shallow sites in CLD3 and CLD4 (*p* ≤ 0.01) but reached only marginal significance or was not significant at deeper sites or in CLD9. Our statistical analysis confirms that DIPC displacement is the most reliable and depth-independent consequence of palmitoylation, while DPPC and CHOL enrichment are depth-sensitive effects most pronounced at shallow palmitoylation sites and diminishing with increasing membrane insertion depth.

Taken together, palmitoylation consistently redirects all four claudins toward ordered lipid domains and positions them at membrane phase boundaries. The magnitude of this effect scales with the extent of depth separation between the two cysteine pairs—greatest in CLD14, intermediate in CLD9 and CLD4, and most modest in CLD3—establishing a quantitative link between palmitoylation site geometry and the degree of lipid-phase reorganization that sets up the mechanistic analysis presented in the following subsection.

### Palmitoylation Depth Determines Lipid-Phase Preference

The phase-boundary localization of all four palmitoylated claudins suggests that the two conserved cysteine pairs do not simply anchor the protein to a single lipid domain but instead orient it across the phase interface. As shown in Figure 5A, the shallow intracellular loop pair consistently points toward the ordered DPPC-rich phase while the deeper C-terminal pair faces the disordered DIPC-rich phase—an asymmetric arrangement that is preserved across all four claudins despite their sequence differences.

**Figure 5:**
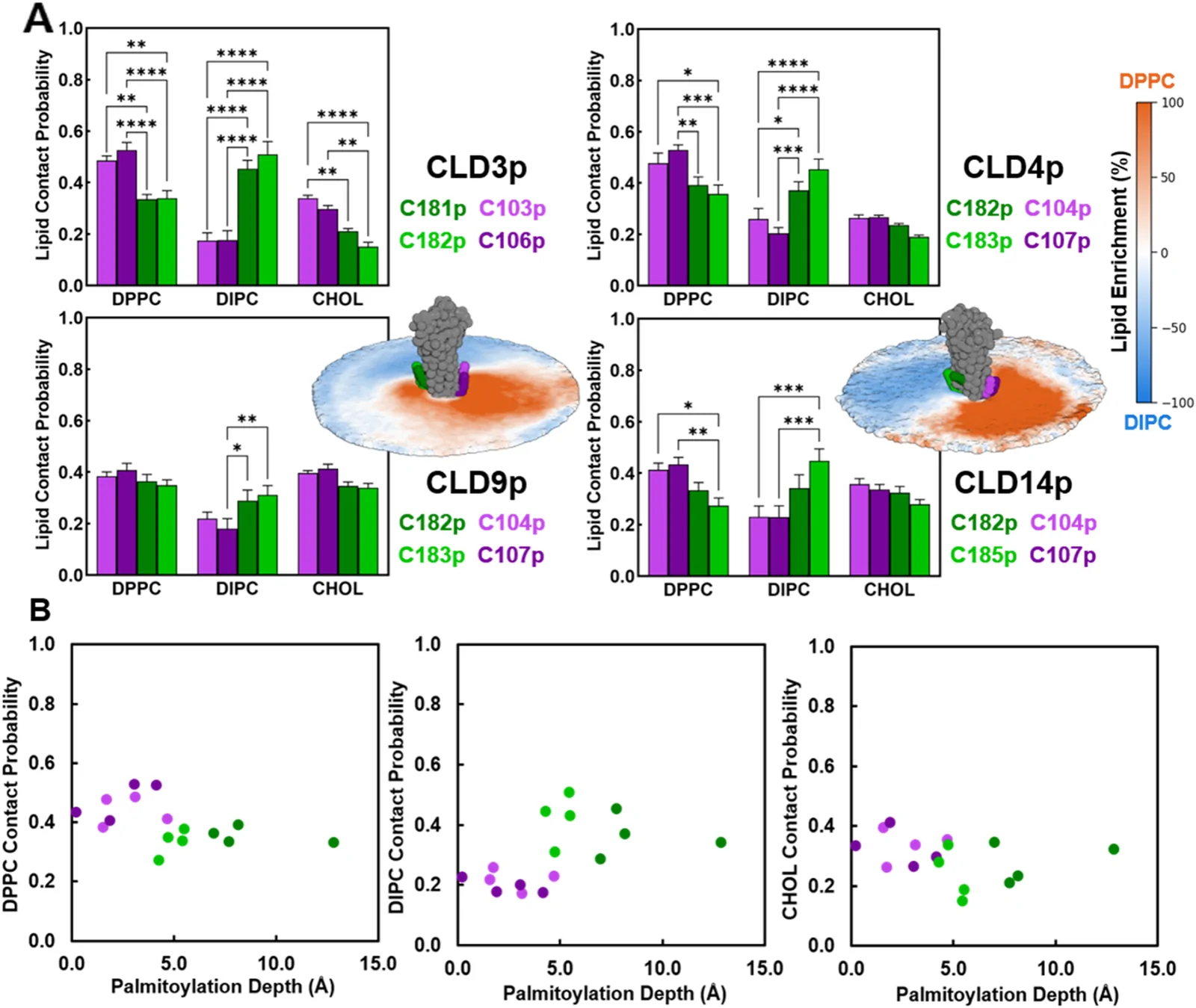
The two pairs of palmitoylated cysteine residues show distinct contact preferences. (A) Lipid contact probabilities for each palmitoylated cysteine residue in CLD3p, CLD4p, CLD9p, and CLD14p, and lipid enrichment maps of the cytosolic leaflet surface shown from an oblique top view. DPPC (orange) and DIPC (blue) distributions around the protein (gray), with colored palmitoylation sites. Bar plots show lipid contact probabilities for DPPC, DIPC, and CHOL colored by the palmitoylation sites. Error bars represent block-averaged standard errors. Statistical significance within each lipid type among the palmitoylation sites was assessed using two-way ANOVA and is indicated as follows: no label (not significant, *p* > 0.05), *­(*p* ≤ 0.05), **­(*p* ≤ 0.01), ***­(*p* ≤ 0.001), and ****­(*p* ≤ 0.0001). The circular shape of the lipid enrichment maps results from the rotational alignment of trajectory frames during the averaging procedure and does not represent the geometry of the simulated membrane. (B) Lipid contact probabilities plotted vs palmitoylation depth for the four claudins. Each point represents one palmitoylated cysteine residue, colored by residue identity.

This asymmetric orientation is reflected in the lipid contact probability data (Figure 5A). Across the claudins, the C-terminal cysteine pair consistently exhibits greater interactions with DIPC and fewer interactions with DPPC and cholesterol than the intracellular loop cysteine pair. This shift in lipid contacts suggests that palmitoylation at the deeper C-terminal sites preferentially promotes localization and stabilization of claudins within the disordered phase. Numerically, CLD3p shows the most pronounced difference between the two site groups: C103p and C106p show significantly higher DPPC and CHOL contact probabilities than C181p and C182p, resulting in a strong reduction of DIPC interactions at the loop sites. CLD4p and CLD14p exhibit a similar but slightly less dramatic trend, where the two cysteine pairs show clear contact differences for both DPPC and DIPC lipids. CLD9p shows a weaker site dependence, with statistically significant differences primarily observed in DIPC contact probabilities rather than in DPPC or CHOL interactions.

Notably, CLD3 exhibits the strongest lipid preference despite having the smallest depth separation between its palmitoylation pairs (Figure 2B), indicating that while relative depth determines the direction of lipid preference within each claudin, the magnitude of the effect also depends on protein-specific structural context. Consistent with this, Figure 5B shows the expected qualitative trend, with shallower cysteines generally favoring DPPC/CHOL and deeper cysteines favoring DIPC. However, this relationship is not quantitatively preserved across all four claudins, as similar absolute depths correspond to a wide range of contact probabilities. Thus, palmitoylation depth is best interpreted as a relative, within-claudin determinant of lipid-phase preference rather than an absolute predictor across the claudin family.

This relationship between depth and lipid preference in claudins presented here has a physical basis in the geometry and packing behavior of the palmitoyl chains themselves. When palmitoyl chains are anchored more deeply within the leaflet, they must tilt or extend laterally to remain within the cytosolic leaflet without crossing the bilayer midplane, a behavior most pronounced in C182p of CLD14p (Figure 6A). This increased conformational disorder reduces their compatibility with the tightly packed ordered lipids and instead favors interaction with the more loosely packed DIPC-rich disordered phase. At shallow embedded depths, by contrast, the palmitoyl chains remain relatively upright with minimal tilting, allowing them to pack efficiently alongside the saturated acyl chains of DPPC and the rigid sterol ring of CHOL in the ordered membrane phase. The positive correlation between palmitoylation depth and palmitoyl chain tilt angle across all residues examined (Figure 6B) confirms that this geometric relationship is systematic rather than incidental to any individual residue or claudin. This geometric relationship supports depth as a structural determinant that precedes and constrains lipid-phase partitioning, consistent with the redistribution toward the ordered phase observed upon introducing palmitoylation at these fixed, sequence-determined sites (Figures 3 and 4). We note, however, that the correlation between tilt and lipid contact probability at a given depth (Figure 5B) does not on its own exclude a reciprocal effect, in which a locally disordered environment encountered by chance could further stabilize a tilted conformation once established; distinguishing these possibilities would require a restrained-geometry simulation, which is beyond the scope of the present equilibrium sampling.

**Figure 6:**
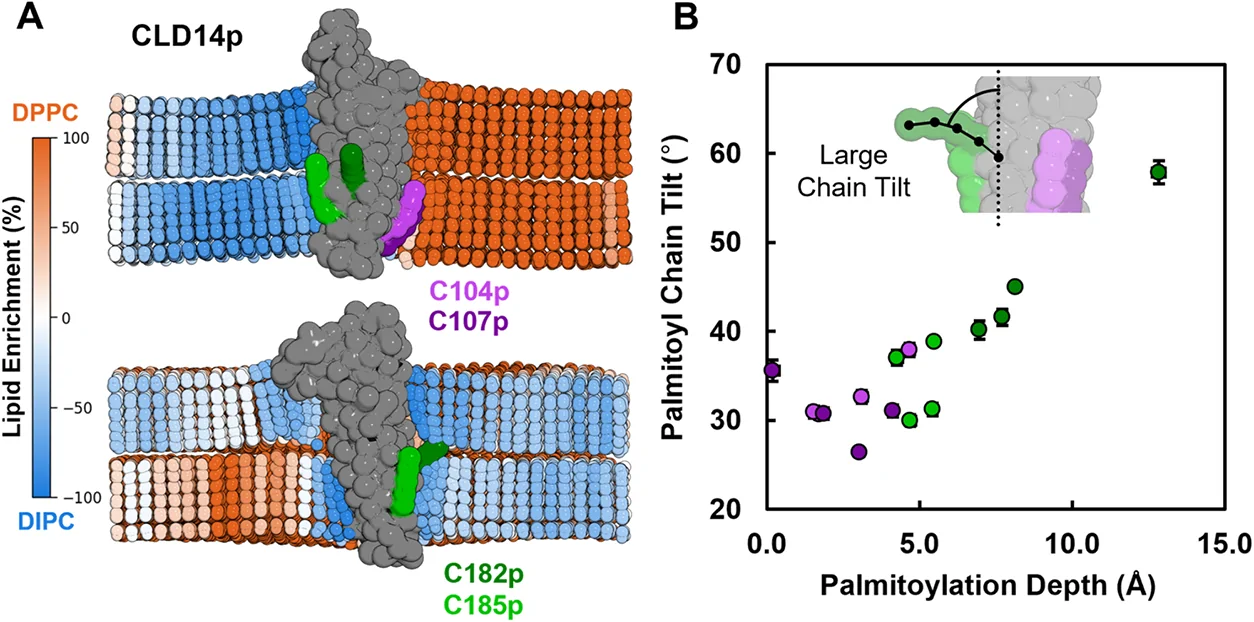
Depth-dependent tilting of the palmitoylated cysteine side chain influences the lipid preference. (A) Cross-section views of CLD14p and surrounding lipid enrichment. The protein and bilayer structure are time-averaged along the simulation. (B) Palmitoylated cysteine side chain tilt plotted vs palmitoylation depth for the four claudins. Each point represents one palmitoylated cysteine residue, colored by residue identity as before. Error bars represent block-averaged standard errors.

Phase-boundary localization of transmembrane proteins, rather than exclusive partitioning into either bulk phase, has precedent beyond claudins. Simulations of the linker for activation of T cells (LAT) transmembrane domain in the same class of DPPC/DIPC/cholesterol ternary system used here have shown a free-energy minimum for protein position located specifically at the domain interface, rather than within either the ordered or disordered phase.^72^ More broadly, a protein whose hydrophobic properties are incompatible with the surrounding ordered phase has been shown to locally nucleate a stable, fluctuating order–disorder interface around itself, an effect attributed to hydrophobic mismatch between the protein and the phase it disfavors.^73^ The mechanism identified here offers a distinct, sequence-encoded route to the same outcome: rather than a single hydrophobic mismatch driving interfacial positioning, claudins achieve it through two spatially separated palmitoyl anchors with opposing phase preferences, without requiring mismatch between the transmembrane domain itself and either phase.

Together, these results show that palmitoylation does not function as a uniform membrane anchor, but as a spatially encoded lipid-sorting mechanism whose output depends on where each palmitoyl chain is positioned within the bilayer: shallow sites drive association with the ordered phase through upright chain packing, while deep sites drive association with the disordered phase through forced chain tilting. Such site-dependent variation in lipid regulation may contribute to claudin trafficking within lipid rafts and orientation between lipid phases; whether it promotes assembly into tight junction structures would require direct investigation of claudin–claudin interactions, which is beyond the scope of the present single-protein simulations.

## Conclusions

By investigating the effect of S-palmitoylation on four claudin family members, our work establishes a direct molecular relationship between palmitoylation and claudin lipid-phase partitioning. Across all claudins examined, palmitoylation increases affinity for DPPC- and cholesterol-rich ordered membrane domains while reducing contact with disordered DIPC lipids. Importantly, we show that palmitoylation does not exert a uniform regulatory effect across all modification sites. Instead, the membrane-embedded depth of the palmitoyl chain determines the lipid-sorting behavior of modified cysteine residues on claudins. Two conserved cysteine pairs within each claudin orient toward opposite membrane phases and exhibit distinct lipid contact profiles, a consequence of depth-dependent palmitoyl chain geometry: upright packing at shallow sites favors the ordered phase, while forced lateral tilting at deeper sites favors the disordered phase. This depth-dependent lipid regulation provides a structural explanation for why palmitoylated claudins preferentially localize at membrane phase boundaries rather than partitioning exclusively into either the ordered or disordered domain, and suggests that changes in palmitoylation depth, whether through differential enzyme access, competing post-translational modifications, or sequence variation among claudin family members, could influence claudin positioning within the membrane. Overall, our work establishes that the membrane-embedded depth of palmitoyl modifications governs membrane protein–lipid specificity, revealing palmitoylation as a spatially encoded mechanism for claudin membrane organization. Given that palmitoylation is widespread among signaling, adhesion, and junctional proteins, it is possible that depth-dependent lipid sorting extends beyond the claudin family; testing this will require examining palmitoylated proteins with different site architectures and topologies.

## Supplementary Material



## Notes

Block-averaged results, and representative snapshots for normalized lipid contacts, embedded depths of palmitoylated cysteine residues, order parameters and tilt angles of palmitoylated cysteine side chains, and lipid membrane phase separation (PDF)
