# Supplementary material for: Palmitoylation Depth Determines Lipid-Phase Preference in Claudins
Source: Langmuir. 2026 Oct 29;42(44):32402–12. doi: 10.1021/acs.langmuir.6c03476 (PMC13757943; doi:10.1021/acs.langmuir.6c03476)

Supporting Information

Palmitoylation Depth Determines Lipid-Phase Preference in Claudins

Xuyang Qin and Shikha Nangia*

Department of Biomedical and Chemical Engineering, Syracuse University, Syracuse, NY 13244, USA

*Address for correspondence:
Dr. Shikha Nangia
343 Link Hall
Department of Biomedical and Chemical Engineering
Syracuse University, Syracuse, NY 13244, USA
Phone (315) 443 0571 | Email: snangia@syr.edu
ORCID 0000-0003-1170-8461

Contents

S1. Normalized lipid contact probability around the palmitoylated cysteine residues. S3
S2. Definition of embedded depth for palmitoylated cysteine residues. S3
S3. Order parameters and tilt angles of palmitoylated cysteine side chains..S4
S4. Block-averaged lipid contact results for the palmitoylation sites. S5
S5. Block-averaged embedded depth results for the palmitoylated cysteine residues. S6
S6. Order Parameters and tilt angles results for the palmitoylated cysteine side chains. S7
S7. Evolution of lipid membrane phase separation during the simulation of CLD14p. S8

S1. Normalized lipid contact probability around the palmitoylated cysteine residues.

The lipids in cytosolic leaflet within the 15 Å cutoff around the palmitoylated cysteine residues were selected to calculate the normalized lipid contact probability to evaluate the affinity to different lipids:

$$p_{ij} = \frac{C_{ij}/n_j}{\sum_j C_{ij}/n_j}$$

p_{ij}: normalized contact frequency between lipid j and residue i
C_{ij}: total number of contacts formed between lipid j and residue i
n_j: total number of lipid molecules (j) present in the cytosolic leaflet
j: three lipid species in the cytosolic leaflet (DPPC, DIPC, and CHOL)

The lipid contacts of each palmitoylated cysteine were computed for every frame and subsequently block-averaged using five to six blocks.

S2. Definition of embedded depth for palmitoylated cysteine residues.

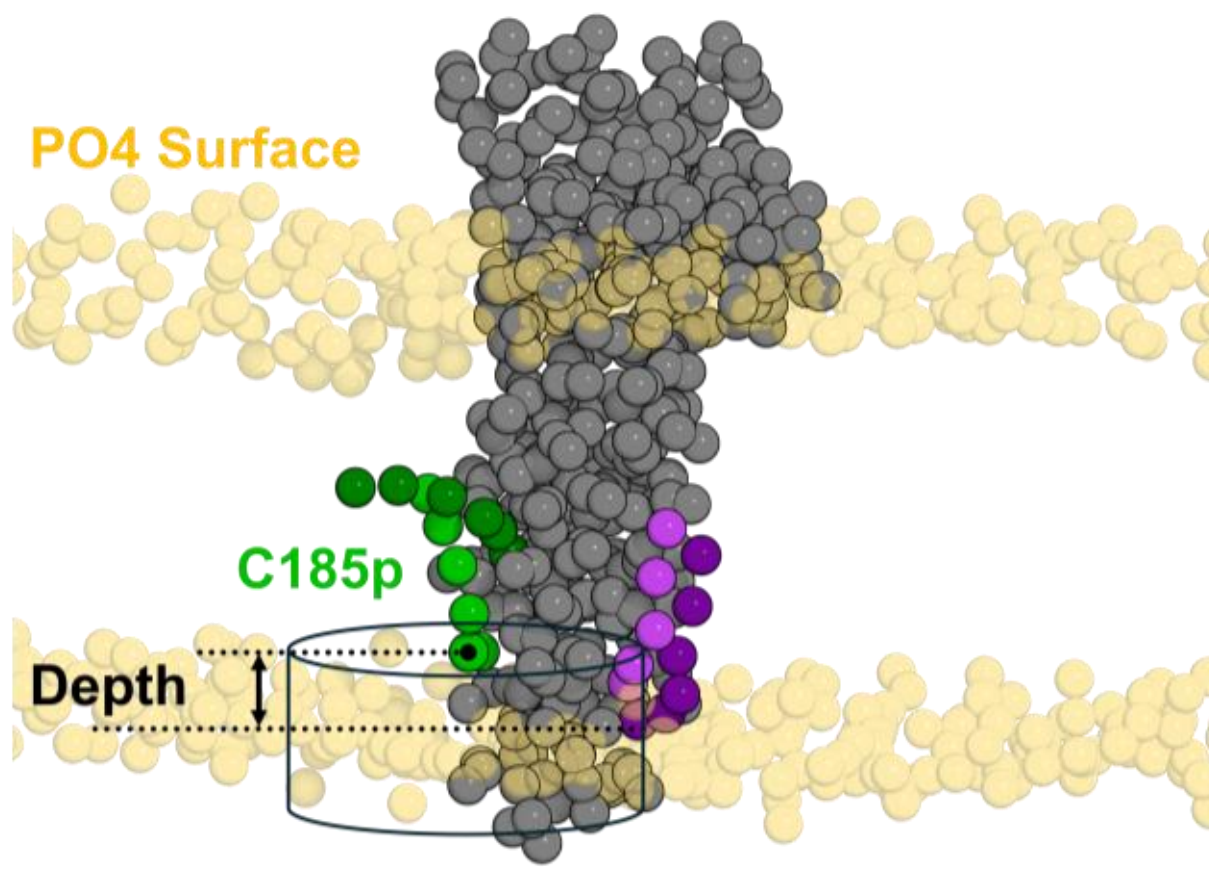

The embedded depth, d, was defined as the average z-coordinate distance between the palmitoylated cysteines' backbone bead (BB) and the surrounding membrane surface (PO4) beads:

$$d = \frac{\sum_i^n (z_1 - Z_i)}{n}$$

d: embedded depth of the palmitoylated cysteines
z_1: z coordinate of BB for the palmitoylated cysteines, $\mathrm{BB}(x_1, y_1, z_1)$
Z_i: z coordinates of PO4 beads in the surrounding region of cytosolic leaflet , $\mathrm{PO4}_i(X_i, Y_i, Z_i)$
n: total number of selected PO4 beads in the surrounding region of cytosolic leaflet.

The surrounding region was defined as a cylindrical zone around the BB in the cytosolic leaflet, by a radius of 15 Å and a z-height of 50 Å. The depth of each palmitoylated cysteine was computed for every frame and subsequently block-averaged using five to six blocks.

S3. Order parameters and tilt angles of palmitoylated cysteine side chains

The order parameters of the palmitoyl chains were computed among the side beads SC1 to SC5 of palmitoylated cysteines using GROMACS. For order in z-axis (S_{zz}), three values were got for each palmitoylated cysteine, and averaged as one to plot and report in the main content.

$$\overline{S_{zz}} = \frac{S_{zz1} + S_{zz2} + S_{zz3}}{3}$$

$\overline{S_{zz}}$**:** mean order parameter along z-axis of the palmitoylated cysteine side chain

S_{zz1}, S_{zz2}, S_{zz3}**:** the three order parameters along z-axis of the palmitoylated cysteine side chain calculated by gmx order

Tilt angle of the each palmitoylated cysteine side chain was converted from $\overline{S_{zz}}$, presenting the tilt relative to z-axis at each frame.

$$\theta_z = \cos^{-1}\left(\sqrt{\frac{2\overline{S_{zz}} + 1}{3}}\right)$$

The full trajectory tilt angles were eventually block-averaged.

S4. Block-averaged lipid contact results for the palmitoylation sites.

Average values and block-averaged standard error are shown in the table below.

		DPPC			DIPC			CHOL		
**CLD3**	**C103**	0.528	±	0.057	0.420	±	0.066	0.052	±	0.012
	**C106**	0.541	±	0.050	0.358	±	0.059	0.101	±	0.010
	**C181**	0.424	±	0.030	0.370	±	0.038	0.206	±	0.009
	**C182**	0.438	±	0.034	0.426	±	0.038	0.136	±	0.008
**CLD3p**	**C103p**	0.486	±	0.018	0.175	±	0.030	0.339	±	0.011
	**C106p**	0.526	±	0.030	0.176	±	0.037	0.297	±	0.014
	**C181p**	0.335	±	0.019	0.454	±	0.033	0.211	±	0.011
	**C182p**	0.339	±	0.030	0.510	±	0.049	0.151	±	0.018
**CLD4**	**C104**	0.431	±	0.065	0.487	±	0.076	0.083	±	0.016
	**C107**	0.456	±	0.068	0.423	±	0.085	0.121	±	0.018
	**C182**	0.304	±	0.055	0.531	±	0.076	0.165	±	0.026
	**C183**	0.302	±	0.056	0.594	±	0.076	0.104	±	0.020
**CLD4p**	**C104p**	0.476	±	0.041	0.260	±	0.042	0.264	±	0.012
	**C107p**	0.529	±	0.020	0.204	±	0.023	0.267	±	0.007
	**C182p**	0.392	±	0.031	0.371	±	0.033	0.236	±	0.007
	**C183p**	0.377	±	0.035	0.433	±	0.040	0.190	±	0.008
**CLD9**	**C104**	0.277	±	0.025	0.443	±	0.044	0.280	±	0.019
	**C107**	0.298	±	0.028	0.459	±	0.050	0.243	±	0.022
	**C182**	0.264	±	0.021	0.453	±	0.035	0.283	±	0.015
	**C183**	0.250	±	0.018	0.480	±	0.030	0.270	±	0.016
**CLD9p**	**C104p**	0.384	±	0.017	0.220	±	0.025	0.396	±	0.009
	**C107p**	0.407	±	0.027	0.180	±	0.039	0.413	±	0.018
	**C182p**	0.364	±	0.027	0.289	±	0.041	0.347	±	0.015
	**C183p**	0.348	±	0.022	0.312	±	0.036	0.340	±	0.016
**CLD14**	**C104**	0.138	±	0.034	0.699	±	0.051	0.163	±	0.019
	**C107**	0.160	±	0.047	0.759	±	0.069	0.082	±	0.023
	**C182**	0.158	±	0.023	0.598	±	0.051	0.244	±	0.029
	**C185**	0.168	±	0.038	0.608	±	0.079	0.224	±	0.039
**CLD14p**	**C104p**	0.413	±	0.026	0.230	±	0.043	0.357	±	0.022
	**C107p**	0.434	±	0.028	0.230	±	0.043	0.337	±	0.019
	**C182p**	0.333	±	0.031	0.343	±	0.051	0.324	±	0.024
	**C185p**	0.273	±	0.031	0.447	±	0.047	0.280	±	0.018

S5. Block-averaged embedded depth results for the palmitoylated cysteine residues.

		Depth (Å)		
**CLD3p**	**C103p**	3.11	±	0.18
	**C106p**	4.13	±	0.11
	**C181p**	7.71	±	0.10
	**C182p**	5.42	±	0.08
**CLD4p**	**C104p**	1.71	±	0.06
	**C107p**	3.04	±	0.06
	**C182p**	8.15	±	0.07
	**C183p**	5.48	±	0.09
**CLD9p**	**C104p**	1.54	±	0.06
	**C107p**	1.87	±	0.11
	**C182p**	6.97	±	0.14
	**C183p**	4.70	±	0.16
**CLD14p**	**C104p**	4.67	±	0.15
	**C107p**	0.19	±	0.21
	**C182p**	12.84	±	0.21
	**C185p**	4.27	±	0.17

S6. Order Parameters and tilt angles results for the palmitoylated cysteine side chains.

The tilt angles were reported with average values and block-averaged standard error.

		S_{zz1}	S_{zz2}	S_{zz3}	$\overline{S_{zz}}$	θ_z
**CLD3p**	**C103p**	0.625	0.619	0.448	0.564	32.6 ± 0.7
	**C106p**	0.731	0.625	0.445	0.600	31.1 ± 0.7
	**C181p**	0.296	0.422	0.300	0.339	41.6 ± 0.9
	**C182p**	0.627	0.664	0.500	0.597	31.2 ± 0.7
**CLD4p**	**C104p**	0.632	0.628	0.568	0.609	30.7 ± 0.6
	**C107p**	0.772	0.749	0.590	0.704	26.4 ± 0.4
	**C182p**	0.306	0.251	0.195	0.251	45.0 ± 0.4
	**C183p**	0.437	0.444	0.349	0.410	38.8 ± 0.4
**CLD9p**	**C104p**	0.614	0.642	0.557	0.604	30.9 ± 0.7
	**C107p**	0.480	0.708	0.636	0.608	30.7 ± 0.6
	**C182p**	0.281	0.470	0.378	0.376	40 ± 1.0
	**C183p**	0.663	0.694	0.520	0.626	30 ± 0.7
**CLD14p**	**C104p**	0.075	0.578	0.648	0.434	37.9 ± 0.8
	**C107p**	0.407	0.487	0.582	0.492	35.6 ± 1.2
	**C182p**	0.000	-0.115	-0.112	-0.076	57.9 ± 1.3
	**C185p**	0.432	0.517	0.420	0.457	37.0 ± 0.8

S7. Evolution of lipid membrane phase separation during the simulation of CLD14p.
Components are color as labeled.

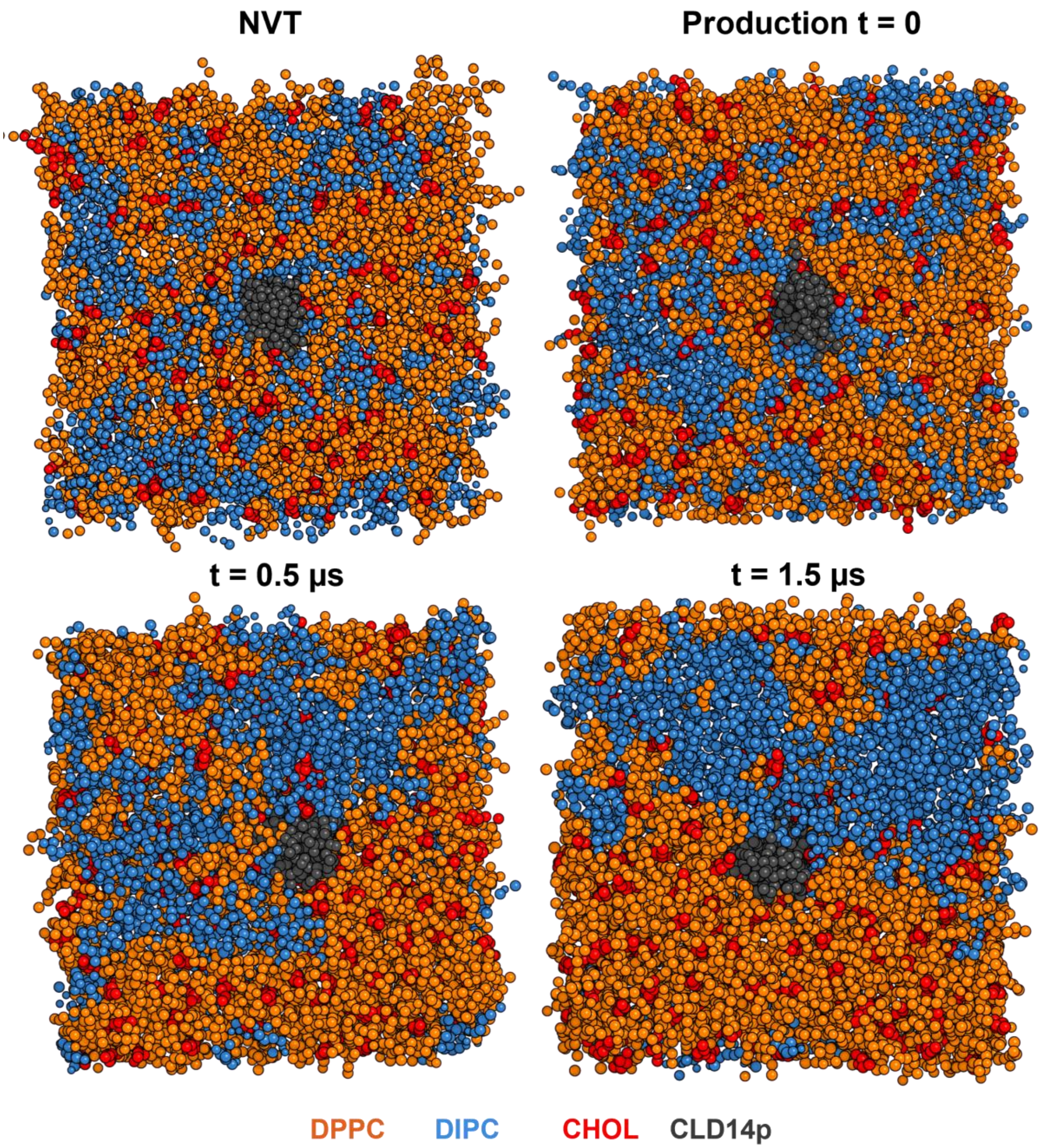

Supplement: Supplementary file 1 [file la6c03476_si_001.pdf]
